# Investigating bioconjugation by atomic force microscopy

**DOI:** 10.1186/1477-3155-11-25

**Published:** 2013-07-15

**Authors:** Ingrid Tessmer, Parminder Kaur, Jiangguo Lin, Hong Wang

**Affiliations:** 1 Rudolf Virchow Center for Experimental Biomedicine, University of Würzburg, Josef-Schneider-Str. 2, 97080, Würzburg, Germany; 2 Physics Department, North Carolina State University, Raleigh, NC 27695-8202, USA

**Keywords:** Atomic force microscopy (AFM), Nanotechnology, Bioconjugation, Nanoelectronics, Nanolithography, Nanomedicine, Biosensors, Nanorobot, DNA origami, Single molecule

## Abstract

Nanotechnological applications increasingly exploit the selectivity and processivity of biological molecules. Integration of biomolecules such as proteins or DNA into nano-systems typically requires their conjugation to surfaces, for example of carbon-nanotubes or fluorescent quantum dots. The bioconjugated nanostructures exploit the unique strengths of both their biological and nanoparticle components and are used in diverse, future oriented research areas ranging from nanoelectronics to biosensing and nanomedicine. Atomic force microscopy imaging provides valuable, direct insight for the evaluation of different conjugation approaches at the level of the individual molecules. Recent technical advances have enabled high speed imaging by AFM supporting time resolutions sufficient to follow conformational changes of intricately assembled nanostructures in solution. In addition, integration of AFM with different spectroscopic and imaging approaches provides an enhanced level of information on the investigated sample. Furthermore, the AFM itself can serve as an active tool for the assembly of nanostructures based on bioconjugation. AFM is hence a major workhorse in nanotechnology; it is a powerful tool for the structural investigation of bioconjugation and bioconjugation-induced effects as well as the simultaneous active assembly and analysis of bioconjugation-based nanostructures.

## Introduction

Bioconjugation of nanoparticles combines unique and orthogonal strengths of two leading edge research fields: the specific interactions of individual biological molecules and novel material properties of nanotechnological compounds. Many of the mechanical, optical, and electric properties of such nanoscale structures are governed by quantum mechanics and open up new options for a wide range of applications. The conjugation with biomolecules can facilitate the controlled assembly of these nanoparticles, as well as modulate their properties or provide them with tags for specific recognition or detection. Biological modifications of nanostructures are increasingly employed in areas as diverse as biodetection, nanomedicine, and nanoelectronics, forming the evolving field of bionanotechnology. The single molecule technique of atomic force microscopy (AFM) offers high sensitivity with nanometer spatial and picoNewton force resolution. Most importantly, AFM is the only imaging platform which allows the monitoring of dynamics of bioconjugates without any labeling modification in physiologically relevant solution and at high temporal (~100 ms) and submolecular spatial resolution [1,2]. Furthermore, combinatory approaches of AFM, such as the combination with optical microscopies or the integration of receptor-ligand recognition detection through bioconjugated AFM tips, further expands the range of simultaneously accessible information on a nanosystem [3-7]. The AFM can also be used as a tool to assemble or manipulate individual bioconjugated nanostructures [3,8]. AFM is hence a major workhorse in nanotechnology; it is a powerful tool for the structural analysis of bioconjugation as well as the effects of bioconjugation on structural and functional properties of nanoparticles. We will try to give an overview over different bioconjugation approaches available to nanotechnology as well as the principle, strength and applications of AFM, in particular with respect to nanostructures. Most importantly, we will then present prominent examples of AFM investigations of bioconjugation of nanostructures and of bioconjugation as a tool in AFM experiments and briefly discuss potential for future developments.

### Bioconjugation as a tool in biological research and nanotechnology

Nature has set us the perfect example of how to elegantly optimize and fine tune different types of processes. The in itself relatively young field of nanotechnology has recently started exploiting the unique strengths of biological approaches. The resulting area of bio-nanotechnology has adopted interaction schemes presented to us by biology, to provide enhanced selectivity, efficiency, or versatility of molecular attachment strategies. Two scenarios of this synergistic scheme are the conjugation of nanostructures as a tool for research in biological science and the conjugation of biological particles as a tool for nanotechnology. For instance, the highly desirable optical properties of quantum dots (QDs), which are nanometer sized semiconductor spheres, make them ideal fluorescent labels in QD-protein conjugates to experimentally follow dynamic protein interactions, both *in vivo* and *in vitro*[9-15]. Biological properties of bio-nanostructure conjugates are, on the other hand, exploited in areas as diverse and as future-oriented as nano-medicine and nano-eletronics. These different areas of interest for bio-conjugated nanostructures will be briefly reviewed (in section Benefits of combining bioconjugation and nanotechnology) following a short overview over different bio-conjugation approaches in nanotechnology (section Biological conjugation strategies).

### Biological conjugation strategies

**Figure 1:**
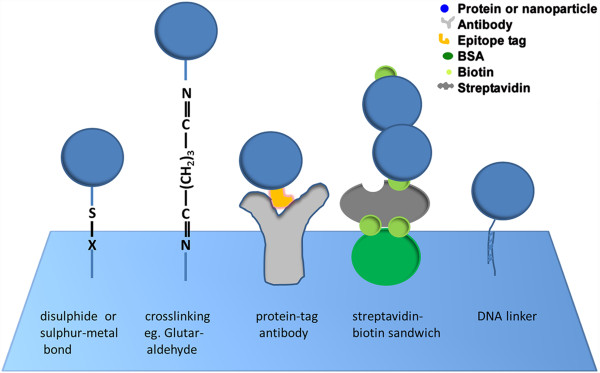
**Different bioconjugation schemes.** From left to right: disulphide bridge (X = S) or sulphur-metal bond (X = metal); chemical crosslinking: for example here the homo-bifunctional crosslinker glutaraldehyde binds an amine group at each end, one on the substrate surface and one on a (protein) molecule to be attached; antibodies that are themselves immobilized on a surface can recognize a specific peptide tag on a protein molecule and can thus serve to tether the protein to the surface; biotinylated bovine serum albumin (bottom dark green oval; biotin in light green) adheres non-specifically to a substrate surface and can anchor streptavidin protein molecules (grey) via receptor-ligand interactions, which in turn can bind biotinylated molecules (blue double circle); particles tagged with single stranded DNA (ssDNA) can be attached to a surface carrying the complementary ssDNA via selective DNA strand annealing.

Functional groups of biomolecules provide a variety of direct or indirect targets for attachment to the (functionalized) surface of nano-structures. Different biological interactions can hereby serve as attachment methods: for example, (i) direct metal-sulfur or disulphide bonds, (ii) crosslinking of functional groups, (iii) antibody linker, (iv) streptavidin-biotin, and (v) DNA complementary base pairing. Bioconjugation approaches have been extensively reviewed elsewhere [16,17]. In the following paragraphs, we will briefly describe general bioconjugation schemes in more detail (see also Figure 1).

(i) Direct metal-sulfur or disulphide bonds

Many bioconjugation applications in nanotechnology involve the attachment of entire protein molecules to the surface of nano-structures. Reactive side chains of amino acids, such as thiol groups (cysteines) or amino groups (lysines) can be used to anchor proteins to these surfaces. In particular, thiol groups can interact directly with surfaces of gold or silver nanoparticles, forming metal-sulfur bonds. These stable interactions can also be exploited to anchor artificially thiolated biomolecules, such as DNA oligomers, to metal surfaces. Alternatively, two thiol (SH) groups (on the substrate surface and on the biomolecule to be attached) can form disulphide bonds under oxidising conditions. The disulphide bonds are, however, weaker conjugates compared to the sulfur metal interaction.

(ii) Crosslinking of functional groups

Specific functional groups in proteins can also be targeted by chemical crosslinking agents. Bifunctional crosslinkers can covalently couple, for instance, primary amines or thiol moieties in a protein with either the same (homo-bifunctional crosslinker) or different reactive groups (hetero-bifunctional crosslinker) introduced on a substrate surface. For example, the homo-bifunctional crosslinker glutaraldehyde bridges two amines, each bound by one of its two terminal aldehyde groups. The length of the crosslinker determines which distance of functional groups in a molecular structure or assembly it is able to interlink. At the same time, the crosslinker provides spacing between the conjugated molecules, which can be desirable (see below). In the case of glutaraldehyde this length or spacing is, for example, approximately 0.7 nm. If required, artificial groups for protein attachment via crosslinking can also be genetically incorporated into proteins [18]. Crosslinkers can take on a variety of forms. For instance, carbodiimide (1-ethyl-3-[3-dimethylaminopropyl]carbodiimide hydrochloride, EDC) catalyzes the direct link between a carboxylic acid and an amine group, without itself being integrated into the molecular structure. Entire polymers (carboxylic acid functionalized polyvinyl alcohol) have been conjugated to protein molecules using carbodiimide technology [19]. The polymers can then, for instance, further act by direct ligand exchange as a capping agent for the preparation of water soluble quantum dots with protein molecules attached to their surface [19]. The recent technology of “click” chemistry is also increasingly employed for the catalysed covalent attachment to reactive groups incorporated into bio-macromolecules, for example via azide-alkyne cycloaddition [16,20].

(iii) Antibody linker

Furthermore, recombinantly expressed proteins can be genetically designed to carry short peptide sequences, so-called epitope tags. A specific tag can be recognized and bound with high affinity by a corresponding antibody, which itself can be bound by a secondary antibody attached to the surface of the targeted nanoparticle. The resulting antibody-sandwich linker structure offers the advantage of larger spacing between an attached protein and the conjugated surface, which can prevent denaturation and/or functional effects on an attached protein by a hard inorganic surface, as presented by most nanostructures [21,22].

(iv) Streptavidin-biotin interaction

The interaction between avidin (or its homologues, such as streptavidin) and its ligand biotin is exceptionally well researched and the strongest receptor-ligand interaction known, with bond strengths of ~200 pN [23]. A further convenient property of this receptor in the context of bioconjugation is that it possesses more than one binding site for its ligand; (strept) avidin can bind up to four biotin molecules. A common surface conjugation procedure in biophysical experiments is based on the strong, nonspecific substrate adhesion of biotinylated bovine serum albumin. A layer of streptavidin molecules can then readily bind to them, leaving free binding sites for further biotin molecules. Biotinylated molecules can thus be selectively and stably bound and immobilized to the receptor molecules attached to biotinylated substrate surfaces via a biotin-(strept)avidin-biotin sandwich structure.

(v) DNA complementary base pairing

The two purines adenosine (A) and guanosine (G) and two pyrimidines cytosine (C) and tyrosine (T) of deoxyribonucleic acid (DNA) polymerize via a sugar phosphate backbone to form a single-stranded DNA (ssDNA) chain. Annealing of two such strands of ssDNA follows the strict rule of A pairing with T (connected by 2 hydrogen bonds) and C paring with G (with 3 hydrogen bonds). The base pairing rule provides selectivity for the annealing of complementary base sequences, while the base-base hydrogen bonds and base stacking add up to form strong contacts between two annealed strands. Contacts between strands with lengths of ≥ 10 base pairs already withstand several (tens to hundreds) pN of force [24]. Annealing of two short complementary single strands of DNA that are attached to different molecules or surfaces can thus be exploited to stably link them.

### Benefits of combining bioconjugation and nanotechnology

The unique physicochemical properties of nanomaterials in combination with the specificity provided by their conjugation to biomolecules open a versatile spectrum of powerful applications. For instance, such hybrid systems have been utilized to identify biomolecular interactions, as transport vehicles in nanomedicine, to track biomolecules optically in real time, and as highly sensitive molecular sensors.

Metal based nanomaterials such as gold nanospheres offer the advantages of low cytotoxicity, high photothermal conversion rate, and photostability [25]. Furthermore, thiolated molecules can directly be attached to the surface of gold nanoparticles [15]. Alternatively, nanoparticle surfaces can be functionalized with, for instance maleimides for attachment of thiol-based ligands [26].

Meanwhile, colloidal semiconductor nanospheres – the so-called quantum dots (QDs) - possess highly desirable fluorescence properties, such as high photostabilities, brightness and quantum yields, as well as excitability in a broad spectral range [11,27]. They have become popular fluorophores, especially in the context of single molecule experiments where strong fluorescence as well as chemical and photophysical stability are highly beneficial for both *in vivo* and *in vitro* experiments [28]. In their original state, QDs are not water soluble, consisting of a semiconductor core, typically CdSe or similar, a thin shell structure of a semiconductor material with a slightly larger band gap, such as ZnS for CdSe cores, and capping ligands for surface passivation (typically trioctyl phosphine/trioctyl phosphine oxide, TOP/TOPO). Solubility in aqueous environment can be achieved via substitution of the TOP/TOPO surface ligands by exposure to an excess of an alternative ligand containing a thiol as well as a hydrophilic functional group, such as mercaptoacetic acid (MAA) [29]. Besides supplying water solubility for the nanoparticle, the choice of reactive group for surface functionalization also allows for conjugation to a variety of different biological targets, such as antibodies or enzymes via disulphide bridges or using crosslinkers. Both metal and semiconductor nanoparticles directly adhere to imidazole carrying substrates, importantly without compromising their optical properties [30]. Alternatively, a polar polymer or peptide capping layer can simultaneously protect QDs against aggressive solution components, induce solubility in aqueous environment, and provide chemical groups for molecular conjugations [31]. A comprehensive overview of different surface modification approaches for quantum dots is presented elsewhere [11]. Conveniently, for quantum dots, most of these surface modifications are already commercially available.

Last but not least, carbon nanotubes (CNTs) and nanowires possess unique mechanical and electrical properties such as quantized energy levels and high, single molecule sensitivity, which are exploited in the development of nanoelectronic components and novel sensing devices. We will provide a brief overview of various specific applications of these different types of nanomaterials in the following sub-sections.

#### Identification and tracking of biomolecules

The unique material properties of nanostructures can be of high interest for the visualization and analysis of biological systems. QDs, gold nanospheres, and carbon nanotubes conjugated to ligand or antibody molecules have been used as labels in microscopy, for instance, to identify cancerous targets inside cells [11,15,32]. However, while QDs offer excellent fluorescent properties, their cytotoxicity is still a problem for *in vivo* applications, where inert gold nanospheres can be good alternatives using dark field illumination microscopy.

#### Biomolecule delivery systems

Artificial organic and inorganic particles, such as metal nanorods [33], carbon nanotubes [34], or even graphene [35,36], also have the potential to become essential carrier devices in nano-medical applications as drug, gene, siRNA, or protein delivery systems. Untreated carbon and graphene nanoparticles have cytotoxic and hydrophobic surface properties [37]. To render them water soluble and biocompatible, their surfaces can be easily functionalized based on established protocols (see also below section AFM can directly visualize bioconjugation) [25,37,38]. Attachment of biological components further allows them to enter the cell via receptor-mediated endocytosis [12,38]. If intended as carrier particles, the load to be delivered can likewise be easily attached to the surface of the nanoparticles. Furthermore, compared to bulk materials, the smaller size and higher surface area-to-volume ratio of nanomaterials enable more efficient loading and delivery of therapeutical agents. Importantly, attachment can be made to be reversible, with controllable release triggered by, for instance, optical or thermal activation to ensure delivery at the desired target [39]. Initial studies have, for instance, shown great promise for modified CNTs in drug and gene delivery, with good cell uptake, tumor suppression efficacies and transduction efficiencies [34,40-42]. In addition, multi-segment nanorods have been demonstrated as good candidates for non-viral gene delivery, as their separate metal components allow for selective multi-functionalization for delivery as well as targeting [33].

#### Bioconjugates in nanoelectronics

The uniquely sensitive electric properties of nanotubes and nanowires make them highly desirable for nanoelectronics applications, for example as nanoelectrodes and nanotransistors in electrochemical devices [43,44]. By attaching peptide nucleic acid (PNA), a nucleic acid analogue with a peptide backbone, to their ends, their assembly can be controlled exploiting the coordination provided by the PNA base complementarity criterion [45,46]. Moreover, PNA or DNA on the nano-structures can be used to arrange them in predetermined nanocircuitry patterns on a substrate surface labeled with ssDNA molecules [47]. Coating with a lipid bilayer can serve to insulate the conducting nanotube or nanowire and further offers optional insertion of proteins forming ion channels or ion pumps. Active ion transport through such ion pumps has been exploited in a nanotransistor set-up to control the source-drain current of the nanotube by the resulting potential build-up [48,49]. While their use as biosensors is still in developmental stages, the electronic and fluorescence properties of carbon nanotubes as well as QDs, coupled – for instance- to glucose oxidase have been shown to sensitively react to the presence of the substrate glucose in a sample solution (see also below, sections AFM can directly visualize bioconjugation and AFM as a nanorobot to manipulate and assemble bioconjugates) [19,50].

### Atomic force microscopy (AFM)

**Figure 2:**
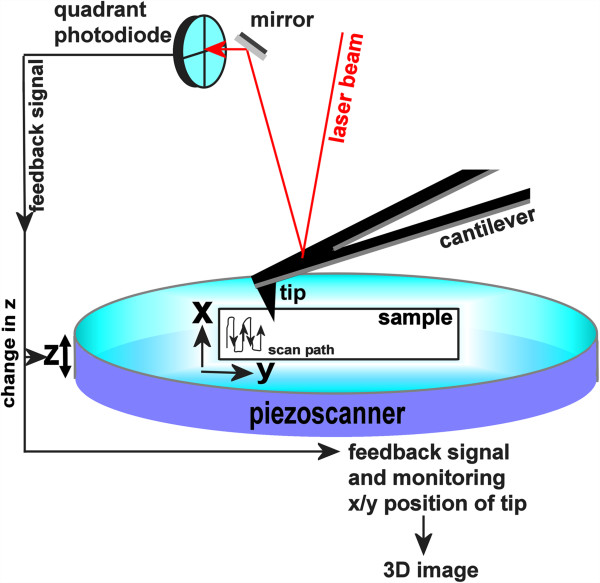
AFM schematic.

Vibrational (i.e. infrared (IR) and Raman) spectroscopy techniques, fluorescence correlation spectroscopy (FCS), and photoluminescence (PL) have been used to monitor the surface chemistry and adsorption processes on nanomaterials based on the bioconjugation induced spectral shifts [19,51-53]. However, successful development and application of bioconjugates demands techniques with nanometer resolution and capacity for monitoring conformational dynamics and nano-manipulation. AFM is an extremely versatile imaging platform, which meets these challenges. In contrast to other, “typical” microscopic techniques, atomic force microscopy (AFM) is a near-field approach, in which the sample surface is directly probed by a needle-like structure, referred to as the AFM tip. For this analysis, the samples are deposited on a substrate surface. In fact, the name atomic force microscopy is highly descriptive of the approach, which measures interaction forces between atoms within the sample surface and atoms within the AFM tip as the tip is brought into contact with the deposited sample (Figure 2). This very different imaging strategy subsequently provides information on very different sample parameters than the far field methods of optical or electron microscopy. Importantly, AFM further achieves very high resolution, comparable with that of electron microscopy and superior to conventional optical approaches.

Tip-sample interactions are combinations from the spectrum of non-covalent forces; long range electrostatic interactions, short range attractive van der Waals forces, and with increasingly close contact the increasing Pauli repulsion of the Lennard-Jones potential. The tip-sample interaction forces are derived from the degree of deflection of a long cantilever arm, at the bottom end of which the AFM tip is mounted (Figure 2). Most AFM systems use an optical system for detection, in which a laser beam is reflected from the back of the cantilever onto a position sensitive photodetector. As the cantilever is bent or deflected towards the surface on attractive tip-sample interactions and away from the surface on repulsive interactions, this results in a positional change of the reflected laser beam on the detector. This change of the laser position on the detector’s quadrant photodiode array is then again translated by the readout electronics into either height information for AFM imaging or an interaction force between tip and sample for AFM force spectroscopy. To convert the measured cantilever deflection into force, knowledge of the cantilever’s spring constant is required (see below, section AFM force spectroscopy). For imaging applications, the cantilever deflection signal is also coupled to the x-y pixel position of the AFM tip, so that a 2-dimensional pixel map of the scanned area then provides a topography image of the sample. In modern commercial systems, a feedback system further constantly re-adjusts the height of either the AFM tip or the sample stage to minimize the forces on tip and sample during scanning.

The two AFM applications, imaging and force spectroscopy, require different experimental approaches and afford different types of information. We will briefly introduce the different requirements and limitations of imaging and force spectroscopy AFM in the following two sections (AFM imaging and AFM force spectroscopy). An in-depth introduction to AFM technology and experimental approaches can further be found, for example, in recent book chapters and reviews [54-59].

### AFM imaging

#### AFM imaging modes

**Figure 3:**
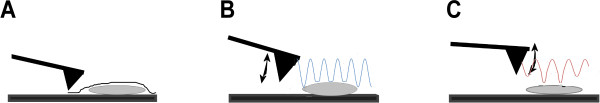
AFM imaging modes: (A) contact mode, (B) intermittent contact, (C) non-contact mode.

Scanning of the sample surface with the AFM tip produces a topographical image of the sample (Figure 2). AFM offers different modes for imaging. The most commonly applied AFM imaging modes are contact mode, intermittent contact (or oscillating) mode, and non-contact mode (Figure 3). While in contact mode the AFM tip directly scans the sample features, in intermittent contact and non-contact mode the AFM tip oscillates above the sample surface. Oscillation is induced by means of a piezo system. The defining difference between the two oscillating modes as well as contact mode imaging is the tip-sample contact. In intermittent contact mode the tip only directly touches the sample at the very bottom of its oscillation amplitude (intermittently), minimizing tip-sample interactions and importantly eliminating lateral forces in the scan process. In non-contact mode, a smaller oscillation amplitude prevents the tip from directly touching the sample surface and only attractive and long range interactions between tip and sample are detected in this mode. Detection in the three different imaging modes exploits different signal parameters. In contact mode, the cantilever deflection is directly translated into height information based on the fact that higher features in the sample bend the cantilever further away from the surface. In the oscillating modes, height information is derived from the change in oscillation amplitude due to tip-sample interactions. In intermittent contact mode imaging, these interactions result in cutting of the oscillation amplitude due to the presence of surface features. In non-contact mode imaging, long range attractive forces are detected by an increase in the tip oscillation amplitude. Detection of these weaker forces in the non-contact mode leads to a gentler imaging process yet mostly poorer image resolution.

In addition to sample height, information on material properties, for instance adhesiveness in the sample, can be derived from its effects on the phase of the tip oscillation. Many nanoparticles also possess interesting material properties such as fluorescence or electric conductivity, which are often ruled by quantum mechanics due to their small size. Such parameters can be accessed by advanced AFM setups with integrated detection of optical, Raman, or electrochemical signals (see below, section Multidimensional AFM approaches).

#### Sample preparation for AFM imaging

Since every feature present on the surface contributes to the images, it is vital to work with pure samples in order to unambiguously interpret the data. For imaging, the sample has to be deposited on a substrate surface. The choice of substrate is dominated by the need for a clean, flat, and smooth surface. Different applications have different requirements. For instance, for imaging at single molecule resolution, often muscovite mica is used, which can easily be cleaved layer by layer to reveal an atomically smooth surface. The mica surface is hydrophilic and negatively charged in near-physiological environment, which can be exploited in the sample deposition strategy. An important criterion for imaging electronic nanocircuitry is substrate cleanliness. The small scale surface roughness of standard AFM substrate materials (such as mica, glass, or silicon) is typically significantly dominated by these samples of semiconductor, metal, or carbon materials due to their height and hardness.

If imaging is carried out in air, the sample is rinsed immediately after deposition on the substrate surface with ultrapure deionized water to remove loosely attached molecules, dried in a gentle stream of nitrogen, and imaged under ambient condition. If a liquid environment is desirable in the experiments, instead of drying the sample, imaging can also be pursued directly in solution. In fact, being able to carry out imaging directly in (near physiological) liquid environment is a major advantage of AFM over, for example, electron microscopy. For experiments in solution, there may be a need to anchor or attach the sample particles to the substrate surface so they are not displaced during the imaging process, depending on the substrate and the sample. This is achieved via surface functionalization with chemical groups to lend it more strongly attractive properties for the sample. For instance, a silicon surface may be rendered positively charged via incubation with (3-aminopropyl)triethoxysilane (APTES), which forms a self-assembled monolayer (SAM) with siloxane bonds to the silicon surface. The amine groups of APTES in these functionalized systems then present a positively charged surface for attachment of sample molecules such as silica nanoparticles or carboxylated carbon nanotubes that are negatively charged under neutral pH conditions.

#### AFM resolution

The high resolution of AFM imaging in the nanometer range is ideally suited for the analysis of bioconjugation processes in nanotechnological applications at the level of the individual molecules. Resolution in the images is limited by the dimensions of the AFM tip as well as by pixel resolution, where these two limiting factors become relevant at different ends of the spectrum of particle sizes. Large objects, such as, for instance, entire bacterial cells or very long nanowires require the scanning and display of relatively large surface areas, with the increasing pixel size determining image resolution. For the imaging of small objects with size on the order of the AFM tip itself or smaller, on the other hand, the sharpness of the AFM tip becomes limiting.The attachment of single molecules of carbon dioxide to the apex of AFM tips has enabled the resolution of individual bonds and transitions in small polycyclic hydrocarbons [60,61]. Non-functionalized commercial AFM tips, however, typically have terminal tip diameters of between 1 nm and 20 nm, which can result in considerable contributions to the apparent dimensions of small particles in the images. Convolution effects of the true sample topography with the geometry of the imaging probe have to be considered and corrected for to get an estimate of the true lateral dimensions of the imaged molecules.This can be done analytically or integrated in the image software when an approximate knowledge of the size of the AFM tip, its radius of curvature, is available. Such information on the AFM tip radius can be obtained by comparison with images of calibration standards [62].

### AFM force spectroscopy

**Figure 4:**
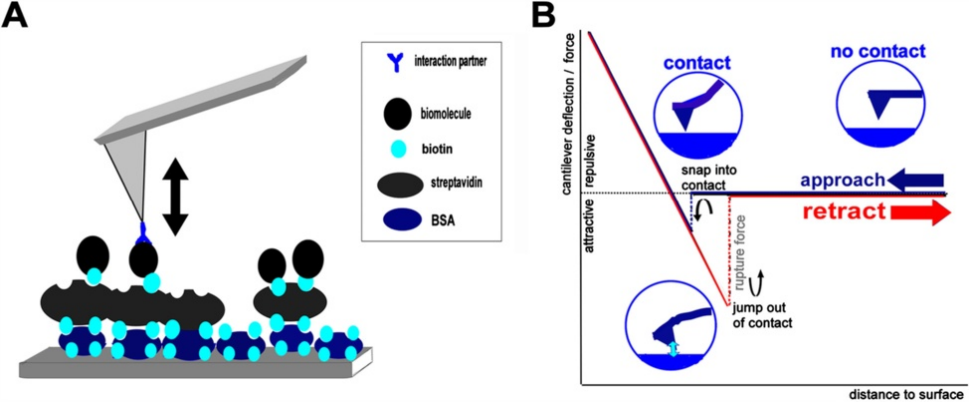
**AFM force spectroscopy. (A)** Sample preparation for AFM force spectroscopy experiments often involves attachment of biomolecules to a substrate surface. Shown here is a streptavidin-biotin sandwich attachment method, in which biotinylated bovine serum albumin (BSA) and streptavidin serve to anchor a biotinylated molecule to a surface. Its interaction partner is attached to the AFM tip and interactions between the two molecules can be monitored from AFM force-distance curves. **(B)** Schematic AFM force-distance curve.

The sensitivity to interaction forces between tip and surface is also exploited in an alternative application of AFM. In AFM force spectroscopy, cantilever deflection *x* in response to tip-sample interactions is measured and translated into an interaction force *F* (Figure 4). Firstly, the cantilever deflection is obtained from the photodetector voltage signal by pressing the tip onto a solid surface and fitting the linearly increasing part of the force curve (Figure 4B). The slope of this line gives us the optical lever sensitivity in units of [V/nm], providing the conversion factor from the measured photodiode voltage to cantilever displacement *x*. Finally, we obtain the interaction force *F* from this cantilever deflection *x* using Hooke’s law, which defines the proportionality constant between the interaction force *F* and the cantilever deflection *x* as the spring constant *κ* of the employed cantilever. *κ* needs to be calibrated for each force spectroscopy experiment. However, modern commercial AFM systems readily provide cantilever spring constant calibration based on measurement of the cantilever’s thermal noise spectrum [63,64].

For measurements of interaction forces or particle elasticity, the molecules of interest are attached to the AFM tip and/or the substrate surface or tethered between tip and surface. For stable attachment of molecules, again the surfaces typically have to be functionalized. Substrate requirements are hence governed by the need to specifically couple or conjugate individual particles to the substrate surface and/or the AFM tip. For example, thiol goups in proteins can form stable sulphur-metal bonds to gold surfaces, while amine groups can be linked to a surface via the bifunctional crosslinker glutaraldehyde or other carboxyl- or aldehyde-based crosslinkers (see above, section Biological conjugation strategies) [65]. In addition to chemical functionalization, often biological molecules serve as a stable link to the surface. For instance, the strong interaction of the streptavidin-biotin receptor-ligand system is a popular aid in molecular attachment (see section Biological conjugation strategies and Figures 1 and 4). Another popular surface immobilization strategy for AFM force spectroscopy is based on the high affinity and specificity of antigen-antibody systems, as is, for example, exploited for the tethering of digoxygenin end-labeled DNA fragments to anti-digoxygenin coated polystyrene beads in force spectroscopy experiments.

AFM force spectroscopy experiments measure the forces between AFM tip and substrate surface from the degree of bending of the cantilever towards the surface. If no interaction occurs during the time of tip-sample contact between molecules on the tip and those on the surface, no forces are exerted on the cantilever during retraction. In this case, the retraction curve resembles the approach curve (Figure 4B). However, if bonds have developed between molecules on the tip and molecules on the surface or if a molecular tether has formed (or pre-existed) to link tip and substrate surface, a force is exerted on the connection between tip and surface during tip retraction. This force increases until at a critical force, termed the rupture force, breakage of the molecular bonds occurs (Figure 4B). We can hence interpret this rupture force in terms of the strength of an interaction.

In dynamic force spectroscopy (DFS), a dynamic spectrum of bond rupture forces as a function of loading rate is used to map the energy barriers traversed along the force-driven pathway, exposing the differences in energy between barriers [66]. For details on the highly complex approach of DFS, the interested reader is encouraged to refer to one of several excellent, extensive reviews on this topic (for example [67,68]).

In the context of testing bioconjugation of nanostructures, force curves can serve as a signature for specific interactions if the rupture force of an interacting system under controlled conditions is known. This signature signal is exploited in applications such as AFM recognition imaging, where a molecular interaction partner is attached to the AFM tip to specifically localize particular molecules in a sample (see below, section Multidimensional AFM approaches). The usefulness of such an identification approach can be envisioned, for instance, for self-assembling monolayers (SAMs) in nanoscale assemblies [69]. Furthermore, such modifications of AFM tip surfaces with biomolecules are exploited for AFM applications as biosensor or nanorobot, machines for the sensitive detection of particle traces in a sample or the molecular assembly, delivery, or preparation of nanostructures [69-71] (see below, sections AFM can directly visualize bioconjugation and AFM as a nanorobot to manipulate and assemble bioconjugates).

### Multidimensional AFM approaches

**Figure 5:**
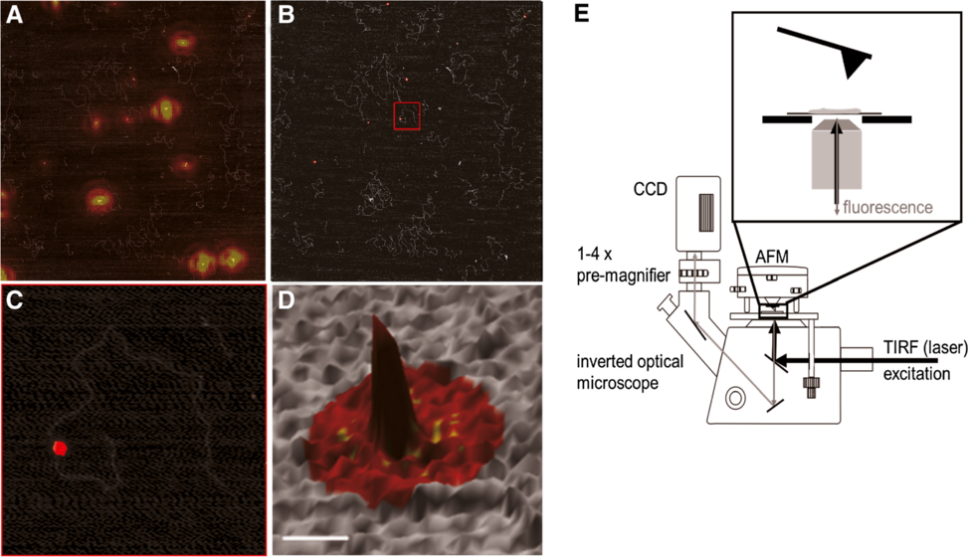
**Combinatory fluorescence-AFM on bioconjugated protein-quantum dot (QD) system.** Reprinted with permission from [14], 2011 Elsevier. **(A)** Registration of raw QD fluorescence signals (yellow-red) with AFM topography (grey scale) of the same sample area (8 × 8 μm^2^). The fluorescence signals were fit by 2D Gaussians to determine their centers with nanometer accuracy, a technique also known as fluorescence imaging with one nanometer accuracy (FIONA). In **(B)**, FIONA signals are shown in red color, indicating localization probability of the fluorescence centers. The red box in **(B)** indicates the QD-protein-DNA complex shown magnified in **(C and D)** as top view and 3D representation, respectively. The scale bar in **(D)** corresponds to 30 nm. These zoom in figures demonstrate good FIONA-AFM overlay accuracy, allowing the identification of a fluorescently tagged molecule in the AFM topography from its fluorescence signal. **(E)** Schematic of Fluorescence-AFM set-up**.** The sample is deposited on a mica substrate (inset zoom, not to scale), excited from below by total internal reflection (TIR) fluorescence (black arrows) and mechanically scanned from above by the AFM. Excited fluorescence (grey arrows) is filtered through a narrow bandwidth emission filter and recorded by a CCD camera attached to the microscope tri-occular port behind a 1× to 4× pre-magnifier.

Combination of AFM with other techniques has opened up a wide spectrum of possible applications. These approaches offer insight into sample topography at high resolution from AFM imaging while at the same time providing information on orthogonal sample properties. Because of the resulting additional level of information, these combinatory approaches are referred to as multidimensional techniques. Conjugated systems of nanoparticles and biological molecules are particularly interesting applications for these multidimensional approaches, since the range of accessible sample properties is significantly increased for these hetero-structures. For instance, labeling protein molecules with quantum dots attaches a fluorescent signal to each of the conjugated molecules. The positions of these fluorescent signals can then indicate and identify the positions of the labeled proteins in the context of complex heteromeric assemblies using simultaneous fluorescence microscopy and AFM imaging (Figure 5) [4,14,72]. Combined fluorescence and AFM microscopy is conceptually straight forward and achieved by simply placing an AFM on top of an inverted optical microscope equipped for fluorescence imaging. The combinatory system can also be used for simultaneous AFM force spectroscopy and fluorescence approaches [73]. Furthermore, such simultaneous applications allow for further improvement of the time resolution of the experiment, exploiting the higher sampling frequency of fluorescence monitoring. Combined fluorescence-AFM set-ups are now commercially available from a number of different AFM companies.

Other multidimensional applications include combined Raman spectroscopy and AFM [74,75] or the simultaneous use of AFM as an imaging tool and as a force sensor. This latter approach involves bioconjugation of the AFM imaging probe itself (see also section AFM as a nanorobot to manipulate and assemble bioconjugates) to achieve specific interactions (recognition events) whenever the AFM tip touches the corresponding partner molecule on the surface. Simultaneous AFM topography and recognition imaging (TREC) results in hybrid images containing sample features as well as the locations of the specifically identified molecules in the sample [69,76].

### AFM and bioconjugation: specific applications

AFM is one of the most versatile imaging platforms. In particular, AFM imaging of nanoparticles in air can serve to directly visualize attachment of biomolecules to their surfaces to follow and confirm conjugation processes and conjugation-induced structural arrangements of nanoparticles. AFM imaging in liquids allows the monitoring of dynamics of conformational changes of bioconjugates in real time with high spatial and temporal resolution. In addition, bio-conjugation applied to AFM probes themselves renders the AFM an active tool in nanotechnology, which can manipulate and modify surfaces and nanostructures. We present examples for these different “passive” and “active” applications of AFM in bio-nanotechnology in the following three sections.

### AFM can directly visualize bioconjugation

Indisputably, bioconjugation of nanoparticles has many applications. As also outlined in section Benefits of combining bioconjugation and nanotechnology, unique properties of the nanomaterials make them useful labels or markers of biomolecules and/or their target sites in biological imaging. The option of controlled release of attached biomolecules has additionally opened up possible applications as delivery vehicles to specific target sites *in vivo*. Finally, changes induced by interactions of proteins can be transmitted to the nanoparticles as used for biosensing [50,77-80]. For most of these applications, it is desirable to know the number of molecules attached to the nanostructure. AFM is a superb method for the characterization of protein-nanoparticle conjugate stoichiometry and functionality.

**Figure 6:**
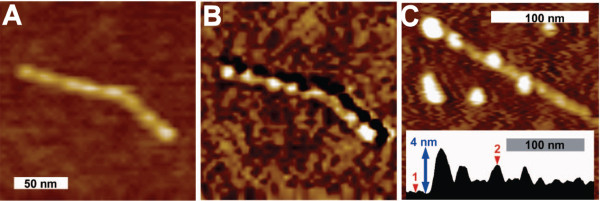
**Wrapping of ssDNA around single-walled carbon nanotubes (CNTs).** DNA wrapping can be seen in AFM topography **(A)** and even better in AFM phase contrast images **(B)**. **(C)** Exposure to end-thiolated DNA results in densely DNA wrapped CNTs that carry regularly spaced functional groups on their surface allowing attachment of mercaptoacetic acid capped QDs (white features on the CNT, nominal QD diameter 2.4 nm) via disulphide bonds . In the section view, (1) denotes a position on the mica substrate while (2) indicates a quantum dot coupled to the CNT. The height scale of **(A)** and **(C)** is 5 nm. Reprinted with permission from [83], coyright 2008 American Chemical Society.

**Figure 7:**
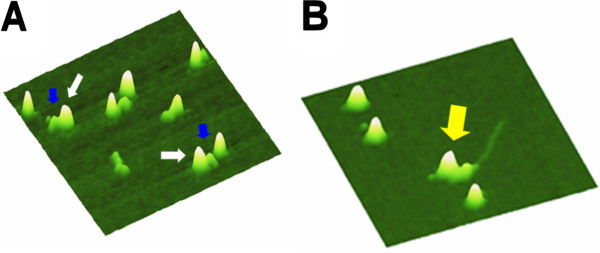
**Functional bioconjugation in a protein-QD system observed by AFM imaging.** Reprinted with permission from [21], copyright 2008 American Chemical Society. **(A)** The arrows indicate single protein molecules of HA-tagged UvrB and primary HA antibody (blue) attached to the surface of a secondary antibody coated quantum dot (white). **(B)** Functionality of QD-conjugated UvrB can be examined from the effect of the conjugation on protein-DNA binding, which is directly visualized by AFM imaging (amount of UvrB bound per DNA fragment). The arrow indicates a QD-protein-DNA complex. Images are 300 nm × 300 nm. Importantly, the relatively large and hard quantum dots (semiconductor spheres with 6 nm core diameter) clearly stand out in the topography. These images thus demonstrate that QDs can serve as a molecular marker to unambiguously identify the presence and location of a labeled protein in AFM images.

Two prominent examples of nanoparticles, for which bioconjugation is of prime interest, are quantum dots and carbon nanotubes. Single-walled carbon nanotubes (CNTs) are versatile nanoparticles, showing interesting mechanical (high strength and flexibility) and electronic (metallic to semiconducting) properties. Their high electrical conductivity coupled with their nanometer size place single-walled CNTs in a unique position for the development of novel electrochemical and electronic devices [43,50,78,81]. For most applications, overcoming the extremely poor solubility of CNTs in aqueous solutions is a prerequisite. This can be achieved by covalent or non-covalent surface functionalization [82-84]. While modifications of the CNT surface with carboxylate groups by oxidizing procedures have been successfully employed for the anchoring of protein molecules via carbodiimide linkages (see section Bioconjugation as a tool in biological research and nanotechnology) [50,78], such covalent functionalization can have adverse effects on the electrical and optical properties of CNTs. For this reason, non-covalent coupling to CNT surfaces is often desirable [82,84]. Non-specific surface coating can be achieved with surfactants or single stranded DNA polymers [83]. Surfactant molecules that adhere to the CNT surface mediate between the hydrophobic surface and the solution. In the non-covalently bound CNT-DNA hybrids, the DNA is wrapped around the nanotubes in a regular pattern, lending hydrophilicity to the system. Topographic and phase AFM images clearly show surfactant induced CNT surface modifications as well as the wrapping of ssDNA around CNTs (Figure 6) [83]. Importantly, the DNA can also directly serve to specifically attach, for example, protein molecules or other nanoparticles, such as gold colloids or quantum dots to the CNT surface (Figure 6). Furthermore, it can be exploited to organize three-dimensional CNT-nanostructures based on DNA annealing or triplex formation [46,83,85,86]. Such superstructures can then, for example, serve as building blocks for nanoelectronic circuits. In addition to being able to analyze the conjugation process itself, AFM imaging allows us to directly visualize these induced superstructural arrangements [46,86] (see also section Solution imaging reveals dynamics of bioconjugates),

Outstanding fluorescence properties of quantum dots (QDs) and electronic properties of CNTs render these excellent means for detection of particles and interactions in single molecule experiments. For instance, as already discussed above, fluorescent QDs can mark the position of a specific protein conjugated to their surface in optical microscopy (Figure 5) [14]. For single molecule studies, to be able to interpret the data correctly, it is essential that each molecule to be studied carry exactly one nanoparticle. AFM imaging allows us to directly, visually analyze labeling stoichiometry at the level of the individual molecules (Figure 7) [21,79]. Close contact to a hard substrate surface can severely affect protein viability and function. It is therefore essential to test effects of the conjugation process on protein activity in order to be able to correctly interpret protein interactions. Functionality of the protein conjugate can be tested by AFM, for instance, by quantifying binding interactions of the protein with and without conjugation to the nanoparticle (Figure 7). Non-covalent protein attachment to hydrophilic (carboxylated) CNT surfaces and antibody sandwich linkages to a QD surface have been shown to retain protein structure and function by AFM as well as spectroscopic approaches [21,82].

Protein attachment can also alter the complex material characteristics of nanoparticles. Potential effects of bioconjugation on quantum dot fluorescence emission can be elegantly and directly investigated in the multidimensional (combinatory) approach of AFM imaging with fluorescence microscopy [14]. Electrochemical sensing experiments, for instance, reveal changes in CNT electron transfer due to protein attachment [50]. This effect on nanoparticle conductivity is exploited in the design of biosensors, in which enzymatic reactions of CNT-coupled proteins can be sensitively detected from the voltammetric response of the hybrid system [50]. For example, immobilizing molecules of glucose oxidase (GOX) on CNT surfaces has been exploited for the sensitive detection of low glucose levels in solution (nanomolar range [77]). GOX is a large dimeric enzyme with monomeric weight of 160 kDa, which catalyses the conversion of glucose to gluconolacetone. Substrate turnover can be detected from the voltammetric response of the CNT electrodes mediated by an induced redox process in the diffusive mediator ferrocene monocarboxylic acid on the carbon surface of the nanotubes [50,78]. In the development of bioconjugated systems for biosensors, a 1:1 stoichiometry is not always necessary or wanted. However, it is still important to know the degree of enzyme loading on the nanostructure in order to be able to calibrate and compare the sensor’s response. Protein coverage of the CNTs can be easily visualized by AFM imaging for the large GOX enzyme molecules [78], but AFM has also been successfully employed for the control of surface immobilization of smaller proteins, such as ferritin (ca. 20 kDa) or even cytochrome c (ca. 12 kDa) [50,78,80]. In an extension of sensory applications of CNTs, the attachment of a single lysozyme molecule to a CNT field effect transistor – as confirmed by AFM imaging- via a pyrene-maleimide linkage allowed for the electric monitoring of protein dynamics with high (microsecond) temporal resolution [79].

Formation of protein multilayers on CNTs is also visible from AFM images. These multiple layers lead to reduced cytotoxicity [82], as is, for instance, highly desirable for applications of CNTs in drug or gene delivery. In this context, AFM has also been applied to visualize protein coated CNTs on cell surfaces [87]. In the future, such imaging studies – especially in combination with fluorescence techniques - may enable us to directly follow CNT uptake by target cells.

### Solution imaging reveals dynamics of bioconjugates

**Figure 8:**
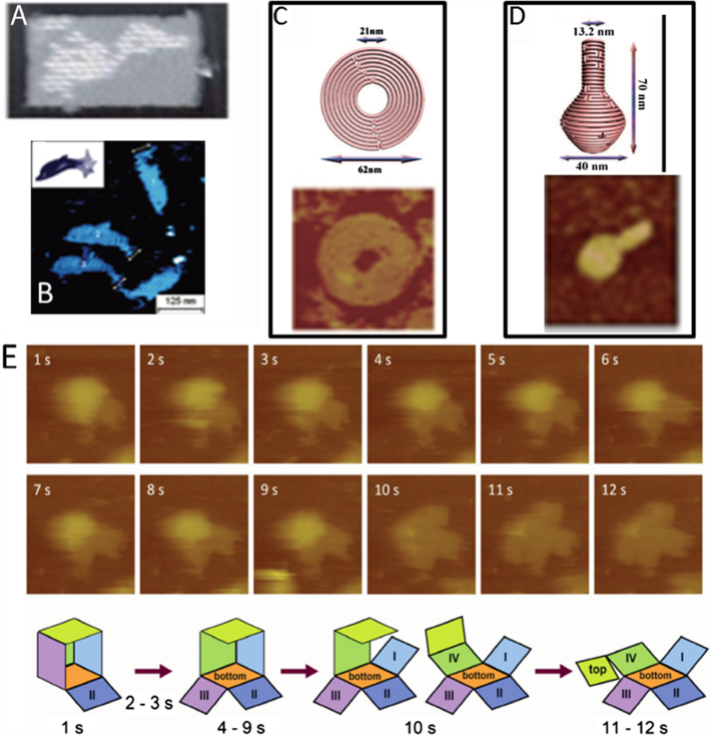
**AFM imaging of structure and dynamics of DNA origami. (A)** An AFM image of DNA origami based map of America. Reprinted with permission from [88], 2006 Nature Publishing Group. **(B)** Dolphin shaped DNA origami structures. Reprinted with permission from [89], 2008 American Chemical Society. **(C)** Schematic drawing (top) and AFM image (bottom) of DNA origami based nine layer concentric ring structure. **(D)** Schematic drawing (top) and AFM image (bottom) of DNA origami based 3D nanoflask structures. **(C)** and **(D)** reprinted with permission from [90], 2011 AAAS. **(E)** High-speed solution AFM imaging and schematic representation of the opening of a 3D origami box with dimensions at 36 nm × 44 nm × 36 nm. Reprinted with permission from [91], 2011 Royal Society of Chemistry.

Solution AFM imaging allows the monitoring of dynamics of conformational changes at high spatial and temporal resolution. For example, AFM studies of DNA origami in solution nicely illustrate the power of this technique for imaging bioconjugates under physiologically relevant conditions [88-91] (Figure 8).

DNA origami is the programmed self-assembly of DNA molecules into intricate contortions, giving rise to highly organized, sophisticated nanometer sized 1D, 2D and 3D structures. When Nadriman Seeman proposed the idea of DNA origami in 1982 [92], he wouldn’t have realized how this simple technique, built on the basis of DNA flexibility, stiffness, and sequence selective self-organization would evolve into an entire multidisciplinary field. DNA origami structures hold promise for numerous applications in areas such as drug release, nanopore sequencing, conformational analysis of biomolecules and nanorobots [93]. Since Seeman first used it in 1998 to image his 2D DNA lattices [94], AFM has become one of the most popular and reliable tools for the characterization of DNA origami, in particular owing to its ability to visualize the molecules in their native environments and at nanometer resolution. In addition, scanning and transmission electron microscopy (SEM and TEM) and high resolution fluorescence microscopies are frequently employed for visualizing DNA origami structural properties. Electron microscopy, for instance, is particularly useful for the imaging of metal-containing DNA origami scaffolds due to the high contrast between the biological and metal materials [95].

DNA origami structures are designed by computer programs. Recent developments in the design programs have enabled the introduction of seam regions in the DNA patterns connected by crossover DNA strands. Solution AFM imaging of the playful design of dolphin shaped DNA origami structures showed control of structural flexibility in the dolphin tails by seam crossover DNA strands [89]. Furthermore, in these experiments, different dolphins could be created that contained recognition sites for each other so that two origami dolphins would “swim together” via intermolecular base pairing, which also resulted in the conformational control of their tails by docking together of these flexible regions. These simple initial constructs point the way to an efficient development of larger and higher ordered structures. Such stabilization with connecting strands – so-called staple strands- as used in the design of the dolphin tails were also utilized to assemble and stabilize 3D structures, for example DNA cuboids [91]. The assembly processes could be directly followed by high speed AFM imaging (Figure 8), the principle and power of which are summarized in a recent review [1]. Recently, controlled origami formation triggered by functionality of dendridic structures attached to the DNA has also been shown by AFM imaging [96]. Kjems and colleagues used AFM in conjunction with other methods to show that the opening of a 3D DNA origami box can be triggered and controlled by light excitation [97]. Importantly, control of the opening of a box structure by a trigger signal is an important step towards powerful applications of such 3D DNA origami structures, for example as accurate drug delivery vehicles. In this context, Jiang and colleagues [98] utilized AFM imaging in a recent study to visualize intercalative drug loading on the DNA, confirming that the DNA origami structures were not affected by the drug loading. This research took advantage of the programmability of the DNA origami nanotechnology to achieve specific drug delivery to selected target sites to circumvent drug resistance. AFM based experiments by Mei and colleagues [99] further indicated that these DNA nanostructures are not only stable but also functional in cell lysates establishing them as candidates for *in vivo* drug delivery and diagnostics.

The rapid and impressive research in this field of nanosystems has further brought about the development of nanomachines that are able to walk on DNA or transport cargo [95,100,101]. Many of these devises can also be controlled by pH, light, or their tracks formed by DNA origami. Exciting developments can be expected in the future, and AFM imaging – increasingly in combination with other microscopic or spectroscopic techniques – will likely be a standard tool for their analysis.

### AFM as a nanorobot to manipulate and assemble bioconjugates

Not only can AFM image and analyze the properties of nanomaterials, but it can also deliver and manipulate molecules at the nanoscale. The term AFM nanorobotics has been coined for this recent advanced application. One of the exciting developments based on AFM nanorobotics is using the AFM tip as a sharp stylus to scratch a substrate surface forming nanopatterns in a nanolithography-type approach. Nanolithographical methods have essential applications in microfabrication, nanotechnology, and molecular electronics. For example, scratching and removing discrete areas in a thiol monolayer on a metal surface and replacing them in solution with thiols terminated by different reactive groups allowed the grafting of an array of fields with different charge or hydrophilic properties [102]. Similarly, surface immobilized protein molecules have been selectively detached using vibrational mode AFM and replaced by alternative proteins from solution [103]. By scratching trenches into a self-assembling monolayer of alkanethiols on gold and immobilizing IgG antibody molecules selectively on these scratched areas, Zhao and colleagues demonstrated the organized assembly of nanotubes, using biological recognition between antibodies on the nanotube surface and the IgG patterns [104]. A rotating-tip-based nanomilling approach has also been successfully employed to remove substrate material in a controlled manner [105]. Moreover, an advanced AFM set-up using two cantilevers was able to perform a pick-and-place motion to move nanowires and arrange them into cross-shaped arrangements [106].

**Figure 9:**
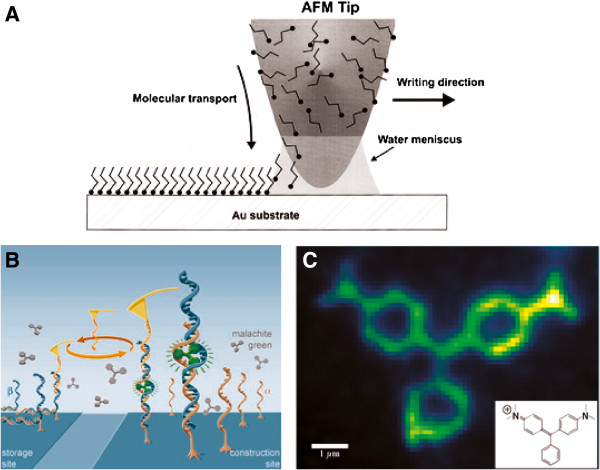
**Dip pen lithography (DPN) and single molecule cut-and-paste technology by AFM. (A)** Schematic representation of DPN. Reprinted with permission from [107], 1999 AAAS. The water meniscus between the AFM tip and the surface serves to transport molecules from the tip to the surface via capillary forces. **(B)** Schematic of the assembly of functional binding sites assembled by the single molecule cut-and-paste approach from individual α- and β-chains of a split malachite green (MG) aptamer. A complete MG binding site was formed by the β-strand transported from the storage site by the AFM cantilever tip and the α-chain at the construction site. For the β-strand transport, the AFM tip was conjugated with handle oligos, which were compatible to the handle sequence at the end of the β-chains. **(C)** Fluorescence microgaph of the final, single molecule cut-and-paste assembled structure, containing more than 500 aptamers. The insert shows the structural formula of the attached MG fluorophore. **(B)** and **(C)** are reproduced with permission from [113], 2012 American Chemical Society.

Attaching molecules to an AFM tip is another approach for delivering them with high, nanoscale precision, using AFM nanorobotics. In 1999, Chad Mirkin and co-workers developed AFM based “dip-pen” nanolithography (DPN) [107]. DPN involves directly “writing” on a substrate surface using molecules as ink. The process uses the AFM tip as a “nib”, a solid-state substrate as “paper”, and molecules with a chemical affinity for the solid-state substrate as “ink”. Molecules are delivered from the AFM tip to the substrate via capillary transport (Figure 9). Since DPN relies on the water meniscus, which naturally forms between the tip and the substrate, tuning the relative humidity can control ink transport rate, feature size and line width. DPN enabled the organization of patterns from two different organic molecules with minute, 5 nm separations in repeated patterning steps [47]. Compared with electron beam lithography, DPN has two major advantages for substrate grafting: because the scanning probe can both generate and locate alignment marks for sample deposition, DPN does not require a resist layer and it is less damaging to the substrate [47]. The same AFM system used for substrate grafting can subsequently be used to analyze successful sample preparation, where both processes profit from the high localization accuracy of the technique [47,108]. Building on earlier studies, DPN-generated nanopatterns have been employed as templates for the organization of semiconductor or carbon nanoparticles [108,109]. Specifically, CNT organization relied on their attraction to the boundaries between hydrophilic and hydrophobic self-assembled monolayer features introduced by AFM based DPN. DPN can also be used directly in liquid environment, as demonstrated by Lenhert and colleagues [110]. In these experiments, the AFM tip was coated with a water insoluble “ink” made of lipids, so that an oil-in-water meniscus formed upon tip-surface contact allowing the lipid ink to be transported to the surface.

Furthermore, conjugation of larger biomolecules to the AFM tip has been used for their direct, precise delivery to specific surface positions. For example, Tang and co-workers utilized the heterobifunctional photocleavable crosslinker succinic acid succinimidyl ester 5-thioyloxy-2-nitrobenzyl ester (SSTN), to functionalize an AFM tip with avidin [111]. When the functionalized AFM tip was approached to a monolayer of biotin immobilized on mica via APTES functionalization, irradiation triggered the release of the proteins from the tip in a photolytic reaction of the crosslinker [111]. The recently developed so-called single molecule cut-and-paste approach surface-assembles a pattern of nanoparticles [112] or organic fluorophores [113] one-by-one and with high precision (Figure 9). Briefly, the approach employs an AFM tip coated with short single stranded DNA oligomers, which picks up DNA strands from a substrate surface and delivers them at a desired surface destination. Both pick-up and delivery are based on a clever combination of force-induced DNA double strand disruption and sequence specific strand hybridization. The surface bound DNA strands finally carry binding sites for the selective attachment of the particle of choice, either being terminated with a biotin moiety for biotin-streptavidin sandwich binding to the surface or via formation of aptamer recognition sequences. The assembled fluorophores could further be directly visualized using a combined fluorescence and AFM system [113]. Such combinatory set-ups have increasingly found use in the direct quality assessment of AFM assembly processes; for instance, Martin Guthold and colleagues have used the AFM to transport fluorescent particles while following their position by fluorescence microscopy [3].

Recently, an AFM-based nanorobot with integrated imaging, manipulation, analyzing, and tracking functions for cellular-level surgery on live samples has been proposed [70]. This augmented reality system also provides a “videolized” visual feedback for monitoring dynamic changes on a sample surface. The nanodevice was shown to be able to deliver epidermal growth factor (EGF) to a cell and subsequently measure the elasticity response of cells contacting the thus stimulated cell.

### Conclusions: future of AFM in bionanotechnology

The power of AFM for the visualization and investigation of bioconjugated nanostructures lies in its high, nanometer resolution capabilities coupled with its ability to image in liquid environment, in which the bioconjugates remain fully functional. The recent advances towards high speed AFM add the invaluable advantage of enhanced time resolution, allowing us to follow many dynamic processes in real time. Furthermore, hybrid AFM applications have demonstrated their unique potentials to simultaneously gain insight on and manipulate bio-nanotechnological constructs. Examples of these are the relatively recent integration of AFM with fluorescence microscopy or combined application of AFM force spectroscopy and topographical imaging. Further advancement and optimization of AFM based platforms with passive observation and/or active manipulation capacities are of great interest for the grand challenge of bioconjugation; to attain an enhanced degree of information on bioconjugated nanoparticles and allow the fine-tuning of bioconjugation to achieve controlled organization of nanostructures.

## Abbreviations

AFM: Atomic force microscopy; APTES: 3-aminopropyl)triethoxysilane; CNT: Carbon nanotube; DPN: Dip-pen nanolithography; EDC: 1-ethyl-3-[3-dimethylaminopropyl]carbodiimide hydrochloride; EGF: Epidermal growth factor; GOX: Glucose oxidase; MAA: Mercaptoacetic acid; PNA: Peptide nucleic acid; QD: Quantum dot; SAM: Self-assembled monolayer; SEM: Scanning electron microscopy; ssDNA: Single stranded desoxyribonuleic acid (DNA); SSTN: Succinic acid succinimidyl ester 5-thioyloxy-2-nitrobenzyl ester; TEM: Transmission electron microscopy; TOP/TOPO: Trioctyl phosphine/trioctyl phosphine oxide; TREC: Topography and recognition imaging; HA: hemagglutinin.

## Competing interests

The authors declare that they have no competing interests.

## Authors’ contributions

All authors contributed to writing and editing of and have read and approved the final manuscript.
